# A High-Content Small Molecule Screen Identifies Sensitivity of Glioblastoma Stem Cells to Inhibition of Polo-Like Kinase 1

**DOI:** 10.1371/journal.pone.0077053

**Published:** 2013-10-30

**Authors:** Davide Danovi, Amos Folarin, Sabine Gogolok, Christine Ender, Ahmed M. O. Elbatsh, Pär G. Engström, Stefan H. Stricker, Sladjana Gagrica, Ana Georgian, Ding Yu, Kin Pong U, Kevin J. Harvey, Patrizia Ferretti, Patrick J. Paddison, Jane E. Preston, N. Joan Abbott, Paul Bertone, Austin Smith, Steven M. Pollard

**Affiliations:** 1 Samantha Dickson Brain Cancer Unit and Department of Cancer Biology, UCL Cancer Institute, University College London, London, United Kingdom; 2 European Bioinformatics Institute, European Molecular Biology Laboratory, Cambridge, United Kingdom; 3 Institute of Pharmaceutical Science, King's College London, London, United Kingdom; 4 Human Biology Division, Fred Hutchinson Cancer Research Center, Seattle, Washington, United States of America; 5 EMD Millipore Corporation, San Diego, California, United States of America; 6 Institute of Child Health, University College London, London, United Kingdom; 7 Genome Biology and Developmental Biology Units, European Molecular Biology Laboratory, Heidelberg, Germany; 8 Wellcome Trust–Medical Research Council Stem Cell Institute, University of Cambridge, Cambridge, United Kingdom; National Cancer Institute, NIH, United States of America

## Abstract

Glioblastoma multiforme (GBM) is the most common primary brain cancer in adults and there are few effective treatments. GBMs contain cells with molecular and cellular characteristics of neural stem cells that drive tumour growth. Here we compare responses of human glioblastoma-derived neural stem (GNS) cells and genetically normal neural stem (NS) cells to a panel of 160 small molecule kinase inhibitors. We used live-cell imaging and high content image analysis tools and identified JNJ-10198409 (J101) as an agent that induces mitotic arrest at prometaphase in GNS cells but not NS cells. Antibody microarrays and kinase profiling suggested that J101 responses are triggered by suppression of the active phosphorylated form of polo-like kinase 1 (Plk1) (phospho T210), with resultant spindle defects and arrest at prometaphase. We found that potent and specific Plk1 inhibitors already in clinical development (BI 2536, BI 6727 and GSK 461364) phenocopied J101 and were selective against GNS cells. Using a porcine brain endothelial cell blood-brain barrier model we also observed that these compounds exhibited greater blood-brain barrier permeability *in vitro* than J101. Our analysis of mouse mutant NS cells (INK4a/ARF^−/−^, or p53^−/−^), as well as the acute genetic deletion of p53 from a conditional p53 floxed NS cell line, suggests that the sensitivity of GNS cells to BI 2536 or J101 may be explained by the lack of a p53-mediated compensatory pathway. Together these data indicate that GBM stem cells are acutely susceptible to proliferative disruption by Plk1 inhibitors and that such agents may have immediate therapeutic value.

## Introduction

Glioblastoma multiforme (GBM) is the most common and aggressive form of primary brain tumour in adults. Current standard of care involves surgery, radiotherapy and adjuvant chemotherapy; however, such treatment regimes fail to provide long-term survival [1]. Our understanding of the biology and genetics of GBM has advanced considerably over the past decade [2]. Concomitant genetic disruptions to the RTK/PI3K, RB/CDK and P53 pathways through point mutations or focal amplifications/deletions are frequent in GBMs [3], [4]. GBM is also accompanied by chromosomal instability with frequent whole-chromosome gains and losses [5]. Gene expression profiling of primary tumour biopsies has indicated at least three major subclasses of disease defined by characteristic marker signatures and associated genetic alterations [6], [7].

GBM tumours display intra-tumoural cellular heterogeneity, with coexistence of distinct subpopulations of cells displaying either neural stem cell-associated markers [8]–[10] or more mature neuronal or glial markers [11], [12]. Stem cell markers can be used to identify cells that are tumour-initiating upon orthotopic xenotransplantation [13], [14]. Thus, the phenotypic cellular heterogeneity in GBMs may reflect an underlying developmental or tissue stem cell hierarchy as originally defined in teratocarcinomas and leukaemias; reviewed in [15].

The molecular and cellular heterogeneity of GBM constitutes an impediment to the identification of a generic therapeutic strategy. One approach to identify molecular vulnerabilities of proliferating tumour cells is to compare their behaviour with normal tissue stem cells in response to chemical/genetic screens. During the past decade improvements in our ability to propagate brain tumour stem cells have been made through application of neural stem cell culture techniques [10], [16], [17]. Neural basal media supplemented with EGF and FGF-2 can support expansion of brain tumour cells that retain stem cell markers and are tumour-initiating [10], [16], [17]. Thus, GBM represents one of the few human cancers where both the genetically normal tissue stem cell and their malignant counterparts can be continuously expanded *in vitro*. These primary cell lines provide more accurate models compared to ‘classic’ serum-derived glioma cell lines which display *in vitro* acquired genetic and epigenetic changes and do not recapitulate tumour cell heterogeneity and infiltration in xenografts [17], [18].

Many investigators have made use of suspension cultures (‘neurospheres’), for *in vitro* expansion of GBM stem cells. However, we and others have suggested that adherent monolayer culture provides a more uniform environment that suppresses spontaneous differentiation and cell death [19]–[21]. Importantly, adherent monolayer culture also permits visualisation by live-cell microscopy of cellular phenotypes at the single cell level, which is a prerequisite for cell imaging-based screening [22].

Kinase inhibitors are the pre-eminent class of therapeutic agents developed by the pharmaceutical industry and many compounds are now in preclinical and clinical development as anti-cancer drugs [23]. Given the spectrum and diverse patterns of structural and mutational changes in GBM, a major challenge is to identify which of the many available molecular targeted therapies should be prioritised for clinical translation. The goal is a therapeutic strategy that can disrupt growth of all subtypes of tumour-propagating cells without affecting normal neural stem and progenitor cell function.

Here we performed a live-cell imaging screen of 160 kinase inhibitors using malignant GNS cell lines and genetically normal human NS cell counterparts. We identify heightened vulnerability to suppression of polo-like kinase 1 (Plk1) across a wide variety of phenotypically distinct GNS cells. This finding suggests that inhibitors of this critical mitotic kinase should be explored as a treatment for GBM.

## Materials and Methods

### Ethics Statement

Human primary cell lines used in this study were obtained according to UK regulations and following approval from the local ethical review board (Lothian Regulatory Ethics Committee; Ref LREC/2002/6/15). Tissue was donated using written informed consent from patients or next of kin for tumour biopsies and foetal tissue, respectively.

### Cell culture of NS and GNS

Human GNS and NS cell lines were derived from glioblastoma biopsies or foetal brain tissues using described protocols [21], [22], [24]. Foetal forebrain tissue from Carnegie stage 23 embryos (∼56 days) was used to establish NS cell cultures. Cells were cultured in serum-free complete medium (CM), N2 and B27 supplemented with Laminin at 1 µg/ml (Sigma) and EGF/FGF-2 (Peprotech, 10 ng/ml) and split typically once per week after dissociation with Accutase solution (Sigma) and centrifugation.

Chromosomal spreads were performed as described [25]. Cells were incubated with demecolcine (0.1 µg/ml, Sigma) overnight and subsequently dissociated, harvested by centrifugation and re-suspended in 5 ml 0.075M buffered KCl and incubated at 37°C for 30 min. Following centrifugation, the pellets were carefully re-suspended in 10 ml of ice-cold fixative (methanol: glacial acetic acid; 3∶1 v/v), incubated at room temperature for 30 minutes, harvested by centrifugation, re-suspended in 1 ml fixative and examined on glass slides. IENS cells were kindly provided by Prof M. van Lohuizen [41]. P53^−/−^ mouse forebrain NS cultures were a gift from Dr P. Dirks.

### Gene expression analysis

The expression of glioblastoma subtype signature genes in GNS cells was examined using published Affymetrix HG-U133 Plus 2 microarray data [22]. Affymetrix HT HG-U133A microarray data for 171 previously used for subtype discovery was obtained from The Cancer Genome Atlas (http://cancergenome.nih.gov/) and used for comparison. Only probesets present on both array types were used for analysis. The two data sets were individually background corrected and normalized with the RMA method in the Bioconductor package *affy* [26]. Gene expression values were computed by averaging normalized intensites of corresponding probesets, using mappings of probesets to genes from Ensembl 67 [27], and centered on the mean across samples. To visualize subtype signatures for glioblastoma samples, expression values were scaled by SD across samples, averaged by subtype, and scaled to units of SD across subtypes.

### Proteomic screening

Cell lines G144 and G166 treated with J101 (100 nM for 1, 2 or 4 h, or DMSO control) were lysed in buffer provided by the manufacturer and tested on a panel of 812 antibodies, 550 pan-specific and 262 phospho-specific (Kinex antibody microarray platform, Kinexus Bioinformatics Corporation). Results from this dataset were filtered using the heuristic *z*-score threshold of ±1.1 on the comparison of treated versus untreated to identify pronounced effects. To study the known interactions between modulated proteins/phospho-proteins, pathway analysis was carried out with Ingenuity software (IPA, Ingenuity Systems). This approach enabled identification of networks significantly enriched for the proteins of interest.

### Chemical screening

Chemical screening was carried out essentially as described in detail elsewhere [28]. Cells were seeded in 96-well plates (3000 cells per well) and left to settle and attach for 3 days. Images were collected at hourly intervals over 3–6 days and included image acquisition prior to addition of the compounds as the time point 0 h. Each inhibitor was added at a final concentration of 100 nM.

A benchtop 96-well format liquid handling device (CyBi-Selma, CyBio) was used to plate cells and dispense chemicals. Images were acquired using the Incucyte HD or Incucyte FLR live-cell imaging system (Essen Biosciences). Initial confluence values typically clustered around 20–30% and final confluence values of DMSO controls around approximately 60–70%.

### Image processing

Incucyte experiments were archived separately. Acquired images were exported as TIF files in the Metamorph ND format with standardised identifiers from which to extract metadata. The CellProfiler image analysis pipeline included loading of images, identification of primary objects, measurement of the imaging area and object size and shape. Data analysis was carried out in R using our in-house developed package ‘*Cellprofile-R*’ (http://code.google.com/p/cellprofile-r/). Example time-lapse sequences were assembled in lossless format using Avid Media Composer. Global brightness and contrast adjustments were applied in Adobe After Effects and video files were rendered in H.264/MPEG-4 from Adobe Media Encoder.

A similar pipeline in CellProfiler was used to quantify DAPI and pHH3 positive nuclei in fixed immunohistochemistry. To characterise the degree of mitotic arrest images of phospho-histone H3-positive cells (*n*≤100) were exported to a thumbnail gallery and classified based on the appearance of the spindle as ‘No spindle’, ‘Unipolar’, ‘Bipolar’ or ‘Multipolar’.

InhibitorSelect 96-Well Protein Kinase Inhibitor Library I and II (EMD, Cat No. 539744) and II (EMD, Cat No. 539745) consists of 160 well-characterised, cell-permeable, potent and reversible protein kinase inhibitors, the majority of which are ATP-competitive. J101 was obtained from Sigma for validation experiments. BI2536, BI6727 and GSK 461364 were obtained from Selleck Chemicals and Imatinib from EMD Merck.

### Quantitative RT-PCR

Total RNA was collected from human and mouse cell samples using QIAGEN RNeasy Mini kit (Qiagen) and cDNA was synthesized with Superscript III (Invitrogen). Quantitative real-time PCR was performed using the LightCycler system (Roche) and data were analysed with the Bioconductor package *HTqPCR* [29]. Samples were normalised to GAPDH or 18S ribosomal RNA for human and mouse samples, respectively. Averages of technical duplicates were used in each experiment. For human samples experimental data were calculated relative to CB660 NS cell expression levels, whereas for mouse samples experimental data were calculated relative to ANS4 cells. PCR primers and associated Universal ProbeLibrary (Roche) sequences are provided in supporting methods.

### Immunocytochemistry and immunoblotting

For immunocytochemistry analysis, medium was carefully removed to avoid cell detachment and cells were fixed with paraformaldehyde 4% for 10 min. After washing with PBS supplemented with 0.1% Triton X-100 (PBS-T), blocking solution with PBS-T+1% BSA+3% goat serum (Sigma) was added for 30 min. Primary antibodies were added to the blocking solution and incubated overnight at 4°C. Primary antibodies used were: goat polyclonal Lamin B (Santa Cruz, sc-6216), rat or rabbit anti-pHH3 (Sigma), mouse alpha-tubulin (Sigma). Appropriate goal secondary antibodies conjugated with Alexa Fluor dyes (Life Technologies) were used throughout.

### Immunoblotting

Cells were lysed in 150 mM NaCl, 20 mM Tris-HCl pH 7.5, 0.5% NP-40, 2 mM EDTA, 1 mM NaF and protease inhibitors. Protein sample buffer was added to lysates and proteins were analyzed by SDS-PAGE followed by wet blotting. The following antibodies were used: anti-α Tubulin (rat monoclonal hybridoma supernatant, kind gift of Dr. A. Hergovich, UCL, London), anti-Plk1 (rabbit polyclonal, Cell Signaling, #4535), anti-Phospho-Plk1 (rabbit polyclonal, Cell Signaling, #5472), anti-Plk2 (rabbit polyclonal, kind gift of Dr. I. Hoffmann, DKFZ, Heidelberg), anti-Sox2 (mouse monoclonal, R&D ab2017), anti-Sox9 (ab3697, rabbit polyclonal, Abcam), anti-GFAP (mouse, monoclonal, Sigma G3893), secondary antibodies ECL anti-rat, anti-mouse and anti-rabbit HRP linked whole antibody (Thermo Fisher).

### Flow cytometry

Unlabelled ANS4 and GFP-labelled 223.2 (p53^−/−^) cell lines were analysed by flow cytometry. In 6-well plates, 50,000 cells of ANS4, 223.2 or a 1∶1 mixture of both cell lines were seeded in duplicate wells. Cells were treated with DMSO, J101 (100 nM) or BI 2536 (100 nM) and at day 3 inhibitors were withdrawn by media replacement. Percentages of GFP-positive cells were determined at days 0, 2, 4, and 6 on the CyAn flow analyser (Beckman Coulter, USA). Data analysis was performed with Kaluza Flow Cytometry software version 1.1 (Beckman Coulter, USA).

### In vitro blood-brain barrier assay

Porcine brain endothelial cells (PBECs) were isolated and cultured as described [30], [31]. Inserts with confluent PBECs were co-cultured with GNS cells and compounds applied to determine their capacity to cross the endothelial barrier to the GNS cells below. After 24 h the cell culture inserts containing PBECs were removed and GNS cells were cultured for a further 24 h, fixed, and then analysed by immunocytochemistry. Sodium fluorescein (Sigma) was used as a control to determine levels of paracellular permeability and therefore integrity of the barrier function of PBECs (applied at 7.5 µg/ml in the top insert). Samples of culture media below the insert were collected and fluorescence was measured at an excitation wavelength of 485 nm and an emission wavelength of 530 nm.

## Results

### Screening of a small molecule kinase inhibitor library identifies compounds that are cytostatic against GNS cells but not normal NS cells

**Figure 1:**
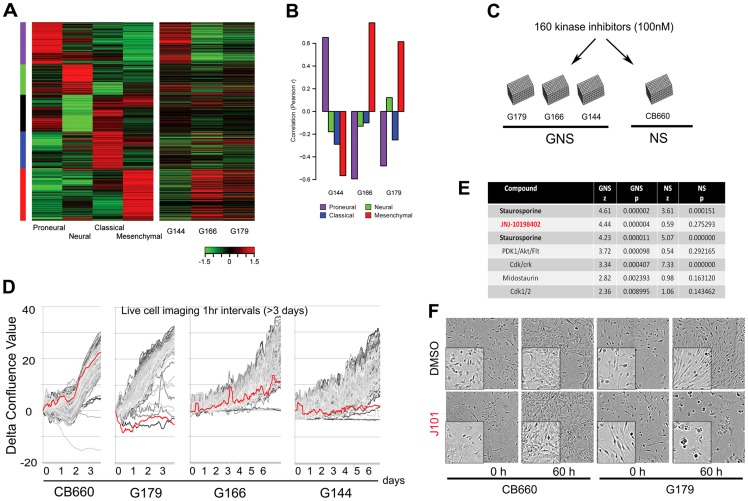
Live-cell imaging screen to determine responses of 160 kinase inhibitors against normal and glioblastoma-derived neural stem cells. (A) Glioblastoma subtype gene expression signatures established Verhaak et al. [7] (left panel) were assessed in a set of GNS cell lines (right panel). (B) Correlations between subtype centroid values determined by Verhaak et al. and gene expression in GNS cells. G144 exhibits clear correspondence to the ‘proneural’ subtype, whereas G166 and G179 have greater similarities to the mesenchymal and neural/mesenchymal subtypes, respectively. (C) Summary of screening strategy based on these three GNS cell lines and a genetically normal NS cell (CB660). (D) Proliferation curves generated for each compound over a 3–6 day period identify J101 (red line) as an agent that can selectively block expansion of GNS cells. (E) Significant events were identified affecting GNS cells but not NS cells, and cytotostatic/cytotoxic compounds reducing confluence in all GNS cells >2.2 standard deviations from the average of DMSO controls are shown (*P* = 0.01). The full data for the screen are presented in Table S1. (F) Example phase contrast images acquired for G179 and CB660 prior to treatment with J101 (0 h) and 60 h.

We previously characterised three glioblastoma-derived NS cell (GNS) lines, termed G179, G166 and G144, with distinct molecular and genetic features [22], [32]. To ascertain whether these correspond to specific molecular subtypes of GBM [7], we assessed levels of the gene expression signatures described by Verhaak et al., comprising 840 genes (Figure 1A and B). We found that G179 and G166 display similarities to the ‘neural/mesenchymal’ and ‘mesenchymal’ subtypes, respectively, while G144 cells express markers associated with the ‘proneural’ subtype [22], [33].

These three primary cell lines were used in a chemical screen to identify small molecules that block proliferation of GNS cells but not normal NS cells. As a control we used a karyotypically normal human foetal NS cell line (CB660) [24]. We utilised a library of 160 known kinase inhibitors (InhibitorSelect I and II, EMD Millipore). This included a broad spectrum of inhibitors of kinases, including tyrosine kinases, cyclin dependent kinases, and protein A, G and C kinases.

Cellular responses to the compounds were monitored each hour over a 3–6 day period using live-cell imaging in 96-well format (Figure 1C). This approach was previously used by our laboratory in a screen of the NIH clinical collection library [22]. Pilot experiments using a 1 µM dose revealed widespread non-specific effects on foetal NS cells (not shown) and prompted us to screen the library at a lower concentration (100 nM). Rates of cell proliferation were extracted from the time-course data by estimating cell confluence at each time point and plotting proliferation curves (Figure 1D). These data were also used to determine a value for the change in confluence between start and endpoints of the treatment (mean of triplicate experiments). Statistically significant events were identified through comparison of library compounds to negative (DMSO) controls for each plate (*P*-values and *z*-score; Figure 1E and Table S1). The pan-kinase inhibitor staurosporine (assayed in two independent wells) served as a positive control and effectively blocked proliferation of all GNS cells and NS cells.

Many compounds had clear cell line-specific effects (Table S1). For example, Cdk/Crk inhibitor was cytostatic against G144 and G166 and stimulated increase in GFAP expression (data not shown), indicating promotion of differentiation, but had virtually no effect on G179. Of particular interest to us was JNJ-10198402 (J101), as this compound disrupted proliferation of all three GNS cell lines, whereas it had no significant effect on the growth of CB660 cultures (*P*<0.00005; *z* = 4.44) (Figure 1F).

### JNJ-10198402 induces mitotic arrest of GNS cells at prometaphase

**Figure 2:**
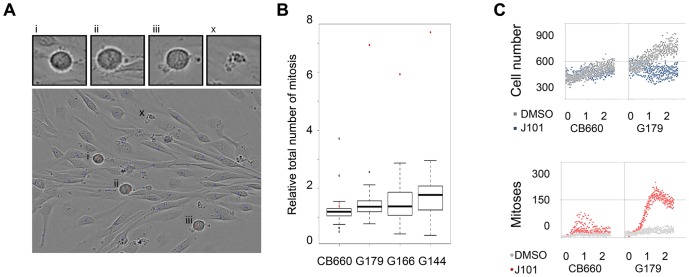
JNJ-10198402 induces mitotic arrest in GNS cells but not in normal NS cells. (A) Segmentation of phase contrast images with high-content analysis software (CellProfiler & CellProfiler Analyst). Objects were assigned into different classes/bins. Interphase cells (blue) and mitoses (red; ‘i–iii’). Erroneously segmented debris or dead cells (green; ‘x’) are isolated and discarded from event counts. (B) Relative number of mitoses scored within 48 h from the start of the experiment for each line and all 160 inhibitors. (C) Kinetics of change in total cell number and mitosis for G179. J101 significantly increased the number of mitoses in G179, but not CB660, without parallel increases in cell number (top panel; blue dots), whereas cells with mitotic morphology increased dramatically during the first 1–2 days (bottom panel; red dots).

The live-cell-imaging screens generated approximately 300,000 image files. These raw data contain a wealth of morphological information regarding cellular responses to each compound. Confluence values generated by the Incucyte system provide only an indirect estimate of cell number, and can be error-prone if morphological changes occur (e.g. flattening of cells). To extract quantitative measurements of cell numbers and morphologies we processed the imaging data with CellProfiler and CellProfiler Analyst (Figure 2) [34], [35]. A principal component analysis (PCA) of the quantitative data for morphological signatures indicated that J101 imposed a unique cellular response in terms of morphology in GNS and not in NS cells (Figure S1).

Individual images were processed using a feature discovery pipeline to segment objects and collect a diverse spectrum of morphological parameters (e.g. size, texture and shape). Using these data we were able to establish prototypes for the identification and subsequent counting of specific cell morphologies. We defined three classes of object/event: 1) cells undergoing mitosis, which have a characteristic rounded morphology with bright halo in the phase channel and visible condensed chromosomes; 2) viable interphase cells; 3) dying cells/debris and cellular processes that were incorrectly segmented [35], [36] (Figure 2A and Movie S1). This analysis provided an independent measure of the total viable cell numbers and relative number of mitosis events within each culture well over the experimental timecourse.

JNJ-10198402 (J101) was confirmed as blocking cell proliferation in GNS cells, as no increases in total cell number were recorded. Surprisingly however, J101 was found to induce a ∼7-fold increase in the numbers of mitotic objects scored during a two-day period compared to DMSO controls (Figure 2B). The increase in cells undergoing mitosis without a concomitant increase in total cell numbers (Figure 2C) suggested that mitotic arrest might be triggered in GNS cultures, but not NS cells, following J101 exposure.

**Figure 3:**
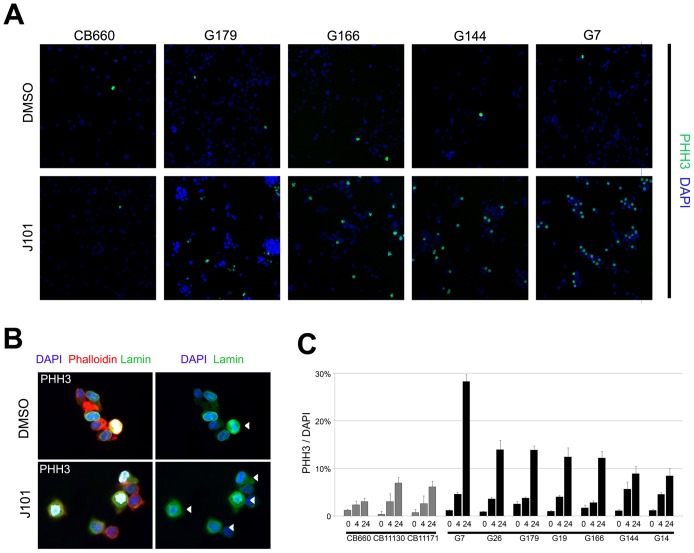
Immunocytochemistry confirms arrest at prometaphase in response to J101. (A) Phospho-histone H3 (pHH3) staining (green) for CB660, G179, G166, G144 and G7 after following treatment with DMSO or J101 (100 nM) for 24 h with nuclear counterstaining using DAPI (blue). (B) Mitotically arrested cells (G7), were immunostained for pHH3 (white), Lamin B (green) and counterstained with rhodamine-phalloidin to visualise actin (red). Arrows indicate the fragmented nuclear membrane, a feature of prometaphase, in mitotic cells as similar in DMSO controls and following inhibitor treatment. (C) Quantification of the ratio between pHH3 stained and total cell numbers (DAPI) from experiments in panel A. Greater sensitivity of GNS cells is observed across seven different cell lines.

Immunocytochemistry for the condensed chromosome marker phospho-histone H3 (pHH3) confirmed that J101-treated cells, but not normal NS cells, had clearly undergone mitotic arrest (Figure 3A). The pHH3-arrested cells displayed a fragmented nuclear membrane, as assessed by co-immunostaining for Lamin B, indicating that arrest occurred specifically at prometaphase (Figure 3B). Manual tracking of individual cells in time-lapse movies revealed that while CB660 engagement in mitosis was transiently prolonged by J101 (2–5 hr), for G144 and G166 we found that cells were often arrested for >10 h, then typically underwent apoptosis (Figure S2). Withdrawal of the inhibitor was not sufficient to enable arrested cells to re-engage in cell cycle, as similar numbers of pHH3 events were measured at 1, 2, 4 and 8 h after drug withdrawal (Figure S3).

We next investigated whether the effects of J101 could be reproduced across a larger set of GNS cells. Four additional GNS cell lines, derived from independent tumour specimens (termed G7, G14, G19, G26) and two newly-derived foetal NS cell lines (CB11130 and CB11171) were tested for responses. GNS cells typically displayed a >10-fold increase in pHH3^+^ cells relative to DMSO controls after 24 h in the presence of 100 nM J101. This represents a 2- to 5-fold increase in the percentage of arrested cells compared to normal NS cell cultures (Figure 3C). Through analysis of gene expression signatures in these four additional GNS lines [7], we found them to mirror features of established GBM tumour subtypes (Johnstone et al., in preparation). G14 and G19 display ‘mesenchymal’ features, while G26 was classified as ‘neural/mesenchymal’; G7, the cell line displaying the most acute response to J101 (Figure 3C), belonged to the ‘proneural/classical’ subtype. Visual inspection of time-lapse movies constructed for G7 revealed that cell division was stalled following entry into mitosis (Movies S2, S3, S4 and S5). These effects were validated on G7 cultures using an independent batch of J101, and by testing the J101 compound synthesized by a different manufacturer (data not shown). In summary, high-content chemical screening identified a kinase inhibitor, J101, that triggers mitotic arrest in a diverse set of GNS cells with limited inhibition of the growth of normal NS cells.

### JNJ-10198402 triggers mitotic arrest of GNS cells through suppression of polo-like kinase 1 and failure to form a bipolar mitotic spindle

J101 (PDGF Receptor Tyrosine Kinase Inhibitor IV, PubChem ID: 9797370) is a cell-permeable indenopyrazole compound acting as an ATP-competitive and reversible inhibitor of platelet-derived growth factor receptors (PDGFRs), with IC50 of 4.2 nM and 45 nM for β and α variants, respectively. To explore whether reduced PDGFR signalling imposed by J101 might influence critical cell cycle regulators, we assessed the transcription factor FOXM1, as its regulatory function is imparted downstream of receptor tyrosine kinase (RTK) signalling pathways. Although levels of FOXM1 were higher in GNS cells than NS cells, we found no change in levels of FOXM1 transcripts or downstream targets Plk1, Aurora, CENP-A, CENP, or p21 upon exposure to J101 (Figure S4). This indicates that J101 acts in parallel or downstream pathways to FOXM1.

Cell-permeable chemical inhibitors can often bind multiple targets, confounding the interpretation of responses and conclusions regarding the critical molecular pathways involved. In addition to PDGFRs J101 also inhibits c-Abl (IC50 = 22 nM) and to a lesser extent c-Kit and several other kinases. We noted that three different PDGFR inhibitors present in the screened library did not phenocopy the effect of J101, even at high doses. Imatinib, which shares a similar target profile to J101, also failed to elicit mitotic arrest (data not shown). This suggests that the observed phenotype might be triggered by inhibition of one or more distinct molecular targets.

**Figure 4:**
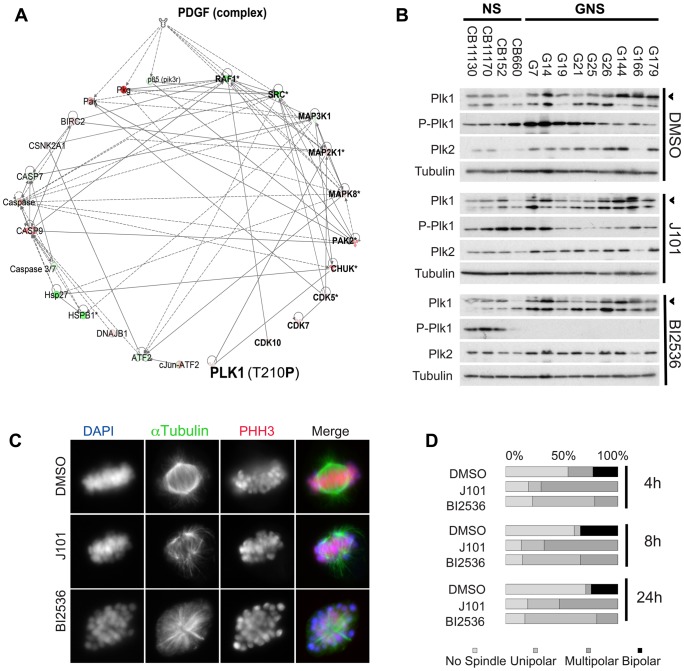
GNS cells arrest at prometaphase due to loss of Plk1 activity and failure of spindle assembly. (A) 812 (550 pan- and 262 phospho-specific) proteins were assessed before and after drug treatment by antibody microarrays. Significant hits are shown and their suggested interactions presented following pathway analysis (Ingenuity). Several kinases (bold) were affected including downstream components of the PDGFR signalling pathway. However, we also noted a suppression of levels of active Plk1 (shown in bold). (B) Western immunoblot for PLK1 and phospho-T210, and PLK2 in a panel of NS and GNS treated with DMSO, J101 or the PLK1 inhibitor BI2536. (C) Immunostaining for spindle protein (α-tubulin) and pHH3 confirms that arrest at prometaphase is associated with aberrant spindle formation. Failure of viable bipolar spindle formation was observed for G7 cells treated with J101 or BI 2536. Photomicrographs show aberrant representative spindles of different morphologies (blue, DAPI; green, (α-tubulin; red, PHH3). (D) Quantification of data from (C) indicates that cells treated with J101 or BI2536 fail to progress to metaphase.

To investigate the key molecular pathways affected by J101 treatment, we used antibody microarrays (Kinexus) to search for changes in levels of 812 proteins and their associated phosphorylated forms. This set represents members of many of the canonical kinase signalling pathways. We assessed G144 at 0, 1, 2, and 4 h time points. In addition to expected changes to downstream components of PDGFR signalling (e.g. RAF1, SRC, p85), we observed a significant decrease in the levels of the active phosphorylated (T210) form of polo-like kinase 1 (Plk1) at each time point (Figure 4A). We then performed kinase profiling, using *in vitro* kinase assays to directly assess the ability of J101 to inhibit activity of 234 kinases (Table S1). This identified Plk1, and its upstream kinase Aurora-A [37], as one of many off-targets resulting from exposure to J101 (Table S2).

The serine/threonine kinase Plk1 is a key regulator of multiple events during mitosis and cytokinesis [38], and is transcriptionally activated by FOXM1 [39]. By western blot we confirmed a reduction of P-Plk1 (T210), but not total levels of Plk1, in response to J101 across a panel of nine GNS cell lines, compared to four independent normal NS lines (Figure 4B). This is likely due to suppression of autophosphorylation and/or a positive feedback circuit (Plk1 is known to phosphorylate itself or upstream kinases such as Auroras). We also noted higher levels of total Plk1 protein in GNS cells compared to the normal NS cell lines.

A reduction of Plk1 activity would likely result in aberrant mitotic spindle formation. To assess spindle morphologies we performed immunocytochemistry for α-tubulin in both DMSO-treated and J101-treated G7 cells. As a control we used the potent and selective Plk1 inhibitor BI 2536 [40], not present in the library originally screened, which suppressed P-Plk1 T210 based on western analysis (Figure 4B). We observed a total lack of viable bipolar spindles or metaphase cells in cultures exposed to J101, with cells often displaying a monopolar or multipolar spindle, similar to BI 2536 treatment (Figure 4D). Thus, GNS cells stall at prometaphase and likely trigger the spindle assembly checkpoint. Similar proportions of abnormal monopolar and multipolar spindles were found at 4, 8 and 24, suggesting this as the primary trigger of cytostasis (Figure 4D). Taken together, these observations lead us to conclude the effects of J101 on GNS cells may be explained by loss of Plk1 activity.

### Low doses of the potent and selective Plk1 inhibitor BI 2536 induce mitotic arrest of GNS cells but not normal NS cells

**Figure 5:**
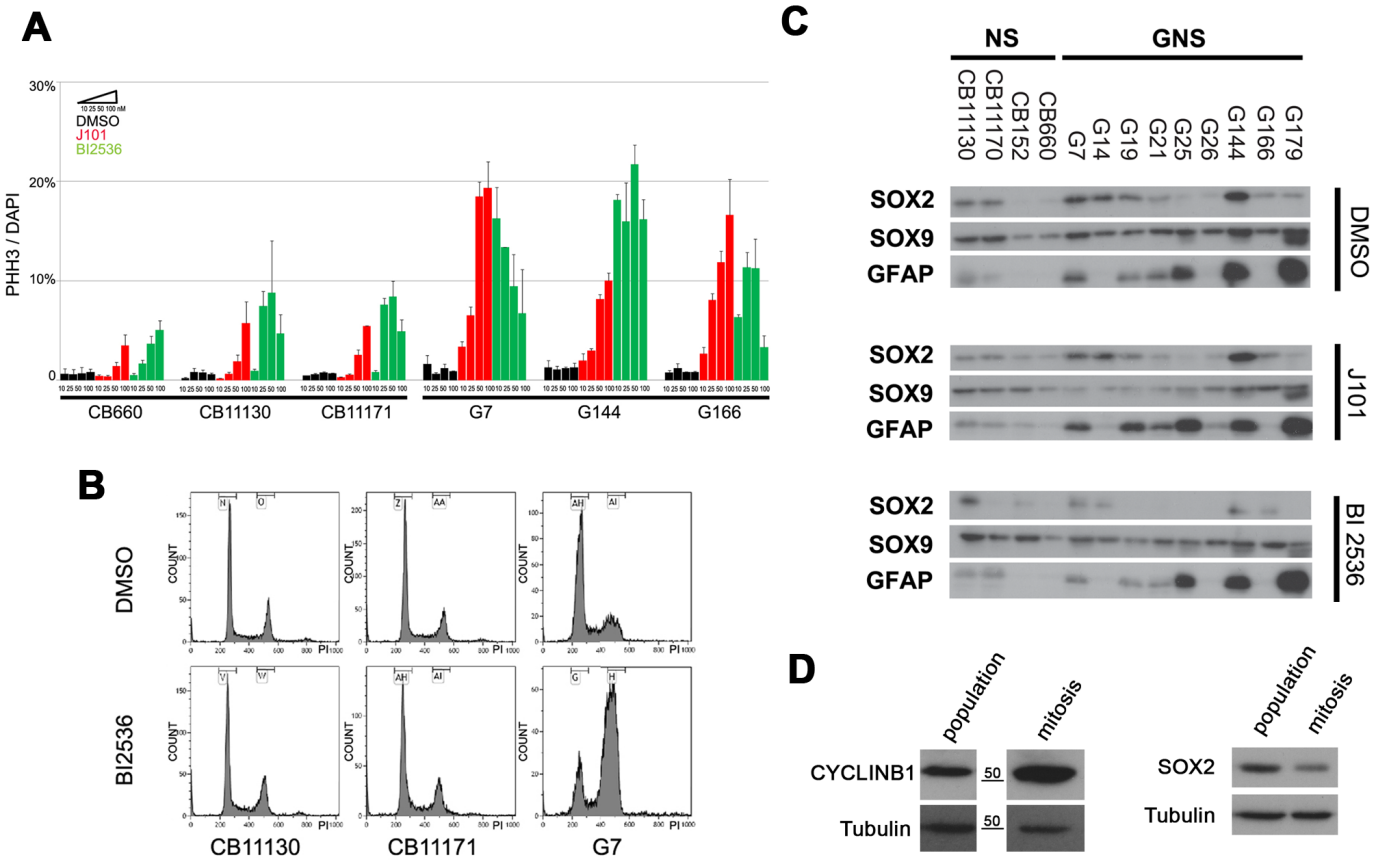
BI 2536, a potent and selective PLK1 inhibitor, disrupts GNS cell proliferation but does not trigger astrocyte differentiation. (A)Dose responses to J101 and BI2536 for three different NS and GNS cell lines, assayed using pHH3 immunocytochemistry 24 h after treatment. (B) Cell cycle profiles of BI 2536-treated NS and GNS cells by flow cytometry confirm a clear increase in the proportion of cells in G2/M for GNS cells, but not normal NS cells. (C) Following BI 2536 treatment, GNS cells display reduced levels of SOX2 but do not have significantly altered levels of the neural stem cell marker SOX9 or upregulation of the astrocyte marker GFAP. (D) Mitotic shake-off was used to enrich for GNS cells in mitosis (confirmed by enrichment for Cyclin B1; left panel). Levels of SOX2 protein were compared in the population as a whole and those undergoing mitosis by immunoblotting. These results suggest a reduction of SOX2 occurs normally during mitosis.

The clinical value of J101 will likely be limited by its lack of specificity. We therefore investigated whether more specific Plk1 inhibitors, such as BI 2536, would also have selective effects on GNS cells while leaving NS cells unaffected. BI 2536 was tested at several doses (10 nM, 25 nM, 50 nM and 100 nM). Of the three GNS cell lines assayed (G7, G144 and G166), all displayed increased sensitivity to BI 2536 at 50 nM, and even 10 nM, when compared to three normal NS cell lines (Figure 5A). This differential response and arrest at G2/M was confirmed by flow cytometry (Figure 5B). We also explored depletion of Plk1 mRNA transcripts by siRNA and confirmed sensitivity of GNS cells to loss of Plk1 activity, albeit less striking than the inhibitor responses, perhaps due to incomplete knockdown (Figure S5).

The effects of BI 2536 on Plk1 were assessed across a panel of GNS cells using immunoblotting. This identified a striking reduction of active Plk1 T210 (Figure 4B), likely due to elimination of a critical positive or autoregulatory feedback mechanism. To explore the potential downstream consequences of mitotic arrest, we examined the neural stem cell self-renewal factors SOX2, SOX9 and the astrocyte differentiation marker GFAP (Figure 5C). It has recently been reported that Plk1 inhibition may promote differentiation of glioblastoma stem cells triggered through loss of SOX2 [41]. Although we did observe a reduction in SOX2 levels following Plk1 inhibition we also found that levels of SOX2 protein decrease normally during mitosis in untreated cells, as assessed following mechanical dissociation and immunoblotting of the mitotically-enriched fraction (Figure 5D). We also did not observe an increase in GFAP expression, or reduction of SOX9 (Figure 5C). Thus, the reduction in SOX2 protein following inhibitor treatment can be explained by the presence of enriched mitotic cells in the population, and is not an indicator of a pro-differentiation response.

**Figure 6:**
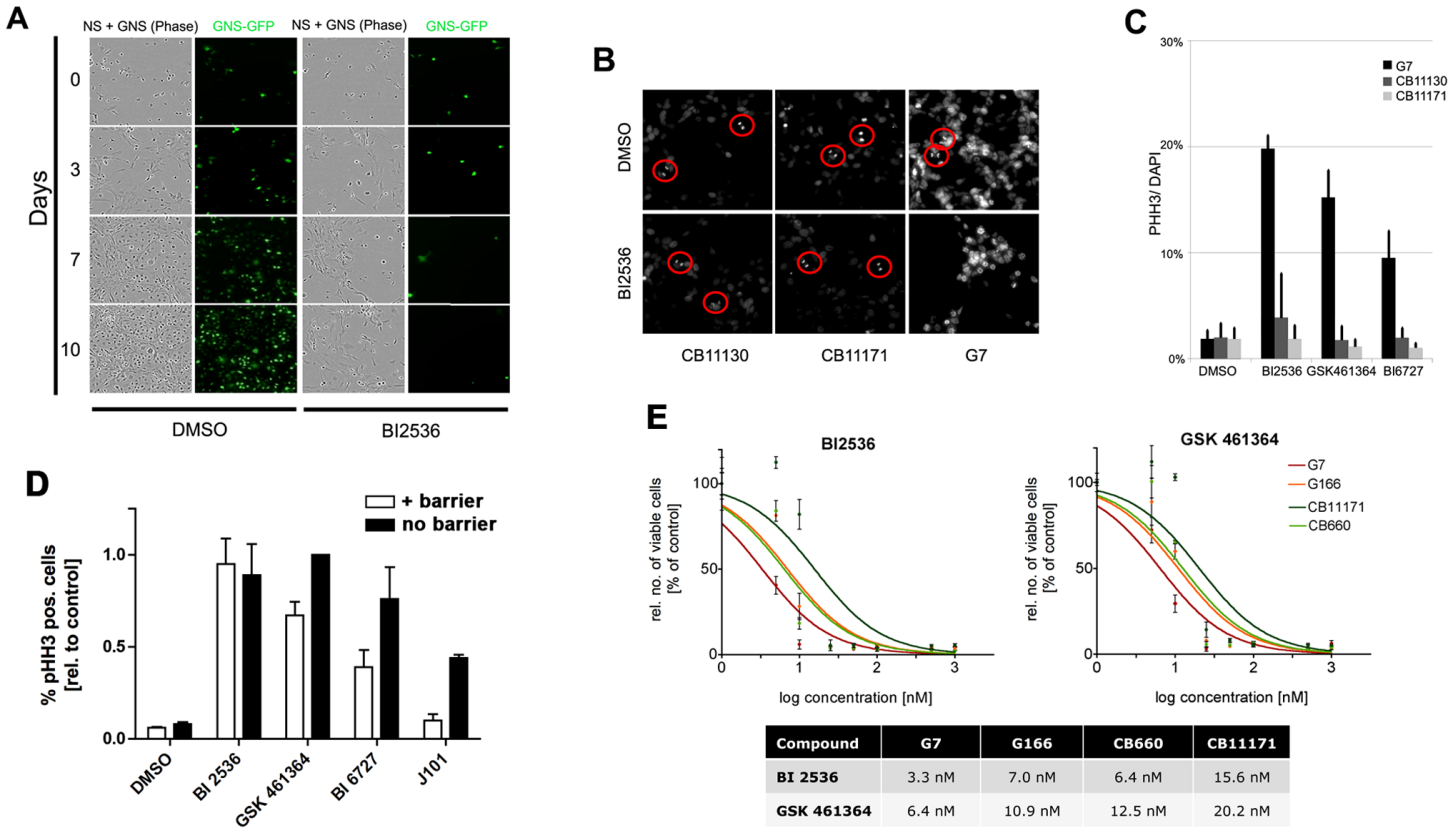
Alternative Plk1 inhibitors in clinical development compare favourably with BI 2536 in selectivity against GNS cells and blood-brain barrier permeability. (A) Co-culture of equal numbers of GFP-transfected G144 cell lines together with wild-type CB660 cells. In the presence of BI2536 GNS cells are selectively lost in the culture, while normal NS cells continue to proliferate. (B) DAPI staining of day 10 cultures of GNS or NS cells confirms that NS cells continue to undergo normal mitosis (anaphase events in red circles), without evidence of mitotic slippage and lagging chromosomes. (C) Two other Plk1 inhibitors in clinical development are selective against GNS cells (G7) compared to foetal NS cells (CB11130 and CB11171; chosen as they have a similar doubling time to G7). (D) Relative numbers of GNS cells arrested in mitosis after 48 h treatment with Plk1 inhibitors using an *in vitro* blood-brain barrier co-culture model. Inhibitors were added directly or via cell culture inserts containing a confluent layer of endothelial cells, and anti-mitotic responses were assessed in G7. J101 displayed poor blood-brain barrier permeability (*P*<0.001). GSK461364 performed similarly to BI 2536. Values shown are percentages of pHH3-positive cells relative to no-insert value of GSK 461364 treated GNS cells (*n* = 3). (E) Dose-response curves and IC50 values for specific Plk1 inhibitors BI 2536 and GSK 461363. NS and GNS cells were treated with different concentrations of the Plk1 inhibitors BI 2536 and GSK461364. Five days after treatment the total number of viable cells was counted and normalised to DMSO control values. Calculation of IC50 values confirmed the differential effect of both inhibitors on NS and GNS cells, with G7 being most sensitive.

To directly confirm the differential responses and viability of normal NS cells using an internally controlled experiment we plated a 1∶1 mix of GFP-transgenic G144 cells together with NS cells treated with 50 nM BI 2536 (Figure 6A). After co-culture for 10 days <5% GNS cells remained, whereas the NS cells had expanded (albeit to a slightly less extent than controls exposed to DMSO at an equivalent concentration). NS lines displayed many viable mitoses and progression through anaphase and telophase after several days without the presence of lagging chromosomes. This suggests that mitotic slippage and subsequent genetic crisis does not occur (Figure 6B).

### Plk1 inhibitors in clinical development induce mitotic arrest in GNS cells and can cross an in vitro blood-brain barrier model

BI 2536 has performed poorly in phase II trials against a range of solid tumours [42]. However, improved Plk1 inhibitors with more favourable pharmacological profiles have since been developed, including BI 6727 [43] and GSK461364 [44]. Each of these inhibitors triggered increased mitotic arrest in GNS cells compared to normal NS cells (Figure 6C).

To stimulate interest in clinical application of Plk1 inhibitors in the treatment of GBM it is critical to establish blood-brain barrier (BBB) permeability. We tested relative BBB permeability for each of the Plk1 inhibitors investigated above, using an established porcine brain endothelial cell (PBEC) BBB model system [30], [31]. PBECs are cultured to confluence on a polycarbonate culture plate insert (filter) which is placed directly above proliferating GNS cells. Inhibitors are then added to the insert chamber and must cross the PBEC layer to affect the GNS cells below.

BI 2536, BI6727, GSK461364 and J101 were tested in the *in vitro* system (Figure 6D). GNS cells affected by the compounds produced the typical mitotically arrested phenotype and were positive for pHH3. As a control the direct influence of the inhibitors on GNS cells without PBECs in the absence of the PBEC-containing inserts was monitored in parallel. The non-specific inhibitor J101 identified in our original screen served as a negative control and exhibited poor permeability (*P*<0.001). Encouragingly, all Plk1 inhibitors tested at 100 nM concentration were able to effectively cross the *in vitro* BBB model and induce a response in the GNS cells. GSK461364 showed comparable efficacy to BI 2536 whereas BI 6727 was less permeant.

To confirm the selective effects of GSK461364 we carried out independent dose-response curves relative to BI 2536. We used the two GNS lines (G7 and G166; which showed the greatest and least sensitivity in earlier experiments, respectively) and two normal NS lines (CB660 and CB11171) (Figure 6E). IC_50_ values of 3.3 nM (BI 2635) and 6.4 nM (GSK461364) were obtained for G7. The NS cell line CB11171 had IC_50_ values of 15.6 nM and 20.2 nM, respectively, confirming differential sensitivity to Plk1 inhibition between GNS cells and NS cells. No significant sensitivity of G166 cells was identified, consistent with our earlier experiments, which was possibly due to the greater levels of total Plk1 protein observed in this cell line (Figure 4B).

### Loss of p53 sensitises NS cells to inhibition of Plk1

Loss of functional p53 signalling is one of the most frequent alterations in GBM and the majority of GNS cell lines contain mutations in *TP53* [45]. Recent analysis of genome-wide expression and somatic mutations in a large cohort of GBMs has identified a putative synthetic lethal interaction between p53 and Plk1 [46], [47]. In addition, several observations in other cancer models have suggested that ablation of p53 may increase dependence on Plk1 activity [48], [49].

**Figure 7:**
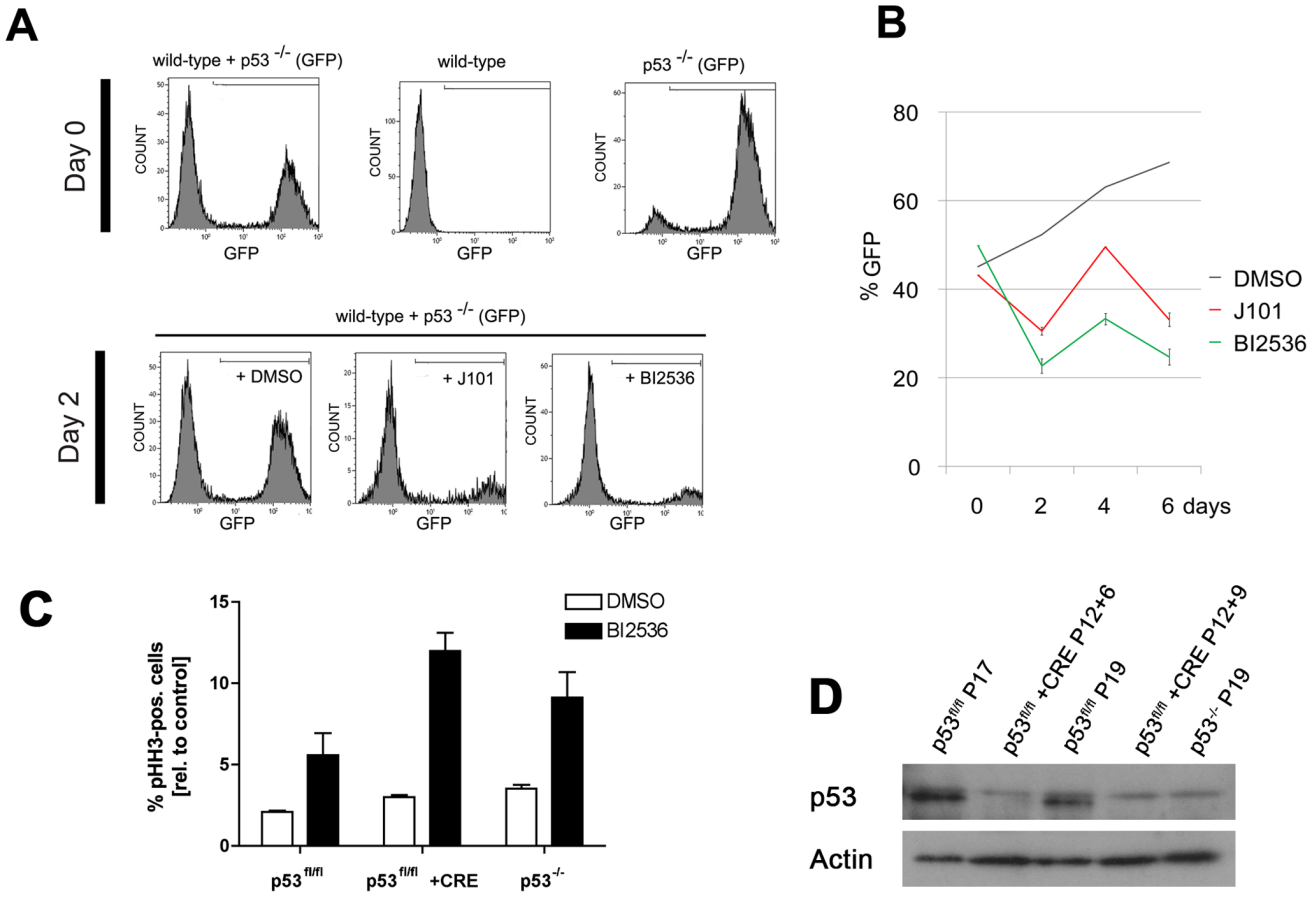
p53 null mouse NS cells display increased sensitivity to Plk1 inhibitors. (A) Wild-type and GFP positive p53^−/−^ mouse NS cells were co-cultured in equal numbers. Both J101 and BI2536 preferentially affect GFP-positive p53 mutant cells. (B) Quantification of flow cytometry data over a 6-day timecourse. (C) Acute genetic deletion of *tp53* sensitizes mouse NS cells to BI 2536 treatment in the absence of aneuploidy. Cells were sensitised to BI 2536 treatment following loss of p53, similarly to an independent p53^−/−^ NS cell line (used in panel A–B). * *P*<0.05. *** *P*<0.001 (*n* = 4; two biological and two technical replicates). (D) Immunoblotting before and after Cre delivery confirms efficient excision of p53 (P = passage number).

To explore whether loss of functional p53 signalling might explain the sensitivity of GNS cells to Plk1 inhibition, we made use of genetically modified mouse NS cells. A p53 mutant GFP-labelled NS line was compared to a wild-type NS cell line for responses to either J101 or BI 2536. We performed a co-culture assay where the two cell lines were plated in equal numbers, and quantified respective populations by flow cytometry. We found that p53^−/−^ cells displayed a greater sensitivity to suppression of Plk1 (Figure 7). A similar result was observed using an independent conditional floxed mutant p53 NS cell line, which displayed increased Plk1 inhibitor sensitivity following acute Cre recombinase-mediated excision of the p53 locus.

Finally, a mouse glioma-initiating NS cell line (IENS cells), lacking INK4A/ARF and overexpressing EGFRvIII [50], also displayed greater sensitivity to BI 2536 or J101 (Figure S6). In contrast to the p53 mutant NS cells, both IENS cells and floxed p53 cells were karyotypically normal (Figure S6). Taken together these results indicate that it is the loss of functional p53 signalling, rather than the aneuploidy inherent to glioma-derived cell lines, that explains the sensitivity of tumour-initiating mouse neural stem cells to Plk1 inhibitors.

## Discussion

Common genetic disruptions in GBM have now been identified and include many of the canonical kinase signalling pathways found in solid tumours. This raises the exciting prospect of exploiting inhibitors developed for other human cancers in the treatment of GBM. In this study we used disease-relevant cellular models of GBM to search for kinase inhibitors that may be prioritized for clinical translation. Our study has identified a sensitivity of GNS cells to inhibition of polo-like kinase 1 (Plk1). This finding complements and extends recent reports indicating a critical importance of Plk1 as a therapeutic target in GBM [41], [46].

GBM is one of the few current examples of a human cancer in which tumour-initiating cells can be expanded continuously *in vitro* and compared alongside normal counterparts. We exploited the experimental advantages and disease relevance of adherent GNS cell lines and were able to compare responses to genetically normal NS cells grown in identical conditions [22], [32]. GNS cells retain hallmarks of distinct disease subtypes, and therefore line-specific molecular sensitivities may help in patient stratification and identification of subtype-specific targeted therapies.

While the identified agent J101 is classified as a ‘PDGFR inhibitor’, our data point to a different molecular target as the likely effector of its cellular response. It remains unclear whether the critical response of J101 is triggered by an effect on Plk1 itself, upstream kinases such as Aurora-A, or a combined effect. We have not explored this further given the same responses are observed using potent and selective Plk1 inhibitors already in clinical development, and also considering the poor performance of J101 in BBB permeability assays.

As reported for other solid cancers, Plk1 protein levels in GBM are higher compared to lower-grade tumours or normal tissues [41], [51]. This is due either to higher proportions of cycling cells in the tumour population or higher Plk1 levels within individual cells. Our data are consistent with the latter explanation. NS and GNS cell cultures have similar doubling times and present no significant apoptosis, yet we observed higher levels of both Plk1 mRNA and protein in GNS cells compared to NS cells. The higher levels of Plk1 in cycling GNS cells may signify an acute dependence on this pathway.

Given the role of Plk1 in many of the key regulatory events during mitosis, we were initially surprised that normal NS cells were able to tolerate reduced Plk1 activity. However, our observations are consistent with previous studies using transformed cell lines [52] and mouse conditional knockdown models, which found no dramatic consequences of Plk1 deletion in normal somatic tissues or cultured fibroblasts [53]. These results suggest a therapeutic window may be defined whereby GBM stem cells are eliminated at a dose that imparts only minor effects on normal somatic cells. It is noteworthy that neuroblastomas, pediatric tumours of the peripheral nervous system, also display greater sensitivity to Plk1 inhibitors [54].

Chromosomal instability is a hallmark of GBM, with frequent whole chromosome gains and losses (e.g. gains of chromosome 7) evident by genetic profiling. Our data using mouse glioma NS cell lines suggests that aneuploidy *per se* is not likely to explain Plk1 sensitivity, but instead point to a role for p53. Altered p53 signalling and RTK/RAS/PI3K signalling pathways are the most common gene alterations observed in GBM (found in 87 and 88% of tumours, respectively) [3]. A consequence of the lack of functional p53 signalling is a widespread upregulation of genes involved in the G2/M checkpoint, including Plk1. This aberration may lead to synthetic lethality [48]. A similar conclusion has been reached for GBM from systems-level pathway analysis of data from the Cancer Genome Atlas project datasets [46]. One possible downstream consequence of the functional p53 pathway in normal NS cells is the increased expression of related family members Plk2 and Plk3, which might compensate for lack of Plk1 and drive cells through mitosis. However, we have found no clear indication that Plk2 or Plk3 are upregulated in response to the inhibitor (Figure 4 and data not shown).

BI 2536 has emerged through phase I trials with adequate safety [55]. However, recent phase II trials failed to report therapeutic benefits for BI 2536 against a wide range of solid cancers [42]. In addition to BI 2536, we found that other classes of Plk1 inhibitor in clinical development (GSK464361 and BI 6727) could also affect GNS cells at low doses. Given the highly infiltrative nature of GBM cells, a critical issue to address for clinical translation of Plk1 inhibitors will be optimisation of blood-brain barrier permeability. Our *in vitro* model demonstrating transit across brain endothelial cells suggests that GSK464361 should be explored further in this regard, with *in vivo* pharmacokinetics and survival benefits in mice harbouring human xenografts across the spectrum of glioma subtypes constituting important future directions.

In summary, combining improved cellular models of GBM and focussed kinase inhibitor libraries with high content imaging-based screening assays, we have been able to identify an acute sensitivity of cells mirroring different GBM subtypes to inhibition of Plk1, likely due to loss of p53 signalling. Given the prognosis for GBM patients, it is now important that those Plk1 inhibitors displaying adequate blood-brain barrier permeability are assessed in clinical trials.

## Supporting Information

Figure S1 **Principal component analysis (PCA) for a set of morphological parameters obtained from the kinase inhibitor screen.** None of the agents in the library, including several PDGFR inhibitors, were able to induce similar mitotic phenotypes. J101 (red dots) was identified in GNS cells but not NS cells as imposing a distinct cellular morphology that was subsequently validated as mitotic arrest. The morphological features of J101-treated cells are distinct from the similar rounded morphology resulting from cell death in response to staurosporine (yellow dots).
(TIF)

Figure S2 **Tracking of individual cells confirms J101 treatment imposes mitotic delays that can lead to mitosis.** Images were obtained at hourly intervals and manually analysed to track individual cells following treatment with DMSO or J101 (100 nM). For NS cells (top), although J101-treated cells were stalled during mitosis, this was resolved after several hours. By contrast, GNS cells (bottom panels) typically arrested at mitosis and eventually underwent apoptosis (see red arrow, frame 14).
(TIF)

Figure S3 **Removal of J101 from GNS cells does not enable progression through mitosis.** GNS cells (G7) were treated with J101 (100 nM) for 24 h. Inhibitor was then removed 0, 2, or 4 h later and cells fixed and stained for the mitotic marker PHH3. Mitotically arrested cells did not immediately proceed through mitosis following drug removal and the majority underwent apoptosis by 24 h.
(TIF)

Figure S4 **mRNA expression levels of FoxM1 downstream targets and related genes in CB660 foetal NS cells (black) and G7 GNS cells (red) as determined by qRT-PCR.** mRNA was harvested after cells were treated with DMSO, BI 2536 (100 nM) and J101 (100 nM) for 5 or 24 h. Data are expressed as fold change relative to CB660 (DMSO). Values are normalised to GAPDH expression. There is no downregulation of FOXM1 transcriptional targets, suggesting the inhibitor lies downstream of FOXM1 activity.
(TIF)

Figure S5 **Transient knockdown of Plk1 mRNA using RNAi.** (A) Four different shRNAs were tested and relative cell numbers were scored. We tested G166 and G7 as these exhibited the least and greatest response to J101 treatment, respectively. For both lines we observed a greater suppression of proliferation in GNS cells (G166 or G7) than normal NS cells. (B) qRT-PCR for Plk1 confirms Plk1 knockdown using these shRNAs.
(TIF)

Figure S6 **Metaphase spreads of mouse mutant NS cell lines.** (A) p53^fl/fl^ cells. (B) p53^fl/fl^ cells transduced with CRE recombinase. (C) p53^−/−^ cells. (D) IENS cells (INK4A/ARF^−/−^ plus EGFRvIII over-expressing NS cells) also display greater sensitivity to Plk1 inhibitors. Genetically normal mouse NS cells (ANS4) were less sensitive than the mouse glioma NS cell (IENS) to both J101 and BI 2536 treated (100 nM each). Cells were treated for 24 h and fixed and immunostained for pHH3. DAPI nuclear counterstain (blue). Sensitivity to Plk1 inhibitors is associated with loss of p53 signalling and occurs in the absence of aneuploidy.
(TIF)

Table S1 **Spreadsheet containing the full data extracted from the inhibitor screen.** Compounds are ranked based on *z*-score.
(XLSX)

Table S2 **234 kinases were assessed for their activity following binding with J101.** Significant off-target effects were observed against Plk1, Aurora-A and Aurora-B (highlighted in yellow).
(XLSX)

Movie S1 **High-content image analysis using CellProfiler and CellProfiler Analyst enables identification and scoring of mitotic cells and total cell numbers.** Example time-lapse movie reconstructed with images processed by CellProfiler Analyst to identify objects as either mitotic (red dots), interphase viable cells (blue dots) or cellular debris/processes (green dots).
(MOV)

Movie S2 **Cellular responses of NS cells (CB660) to DMSO and J101, respectively.**
(MOV)

Movie S3 **Cellular responses of NS cells (CB660) to DMSO and J101, respectively.**
(MOV)

Movie S4 **Cellular responses of GNS cells (G7) to DMSO and J101, respectively.**
(MOV)

Movie S5 **Cellular responses of GNS cells (G7) to DMSO and J101, respectively.**
(MOV)
