# Supplementary figures and images for: A High-Content Small Molecule Screen Identifies Sensitivity of Glioblastoma Stem Cells to Inhibition of Polo-Like Kinase 1
Source: PLoS One. 2013 Oct 30;8(10):e77053. doi: 10.1371/journal.pone.0077053 (PMC3813721; doi:10.1371/journal.pone.0077053)

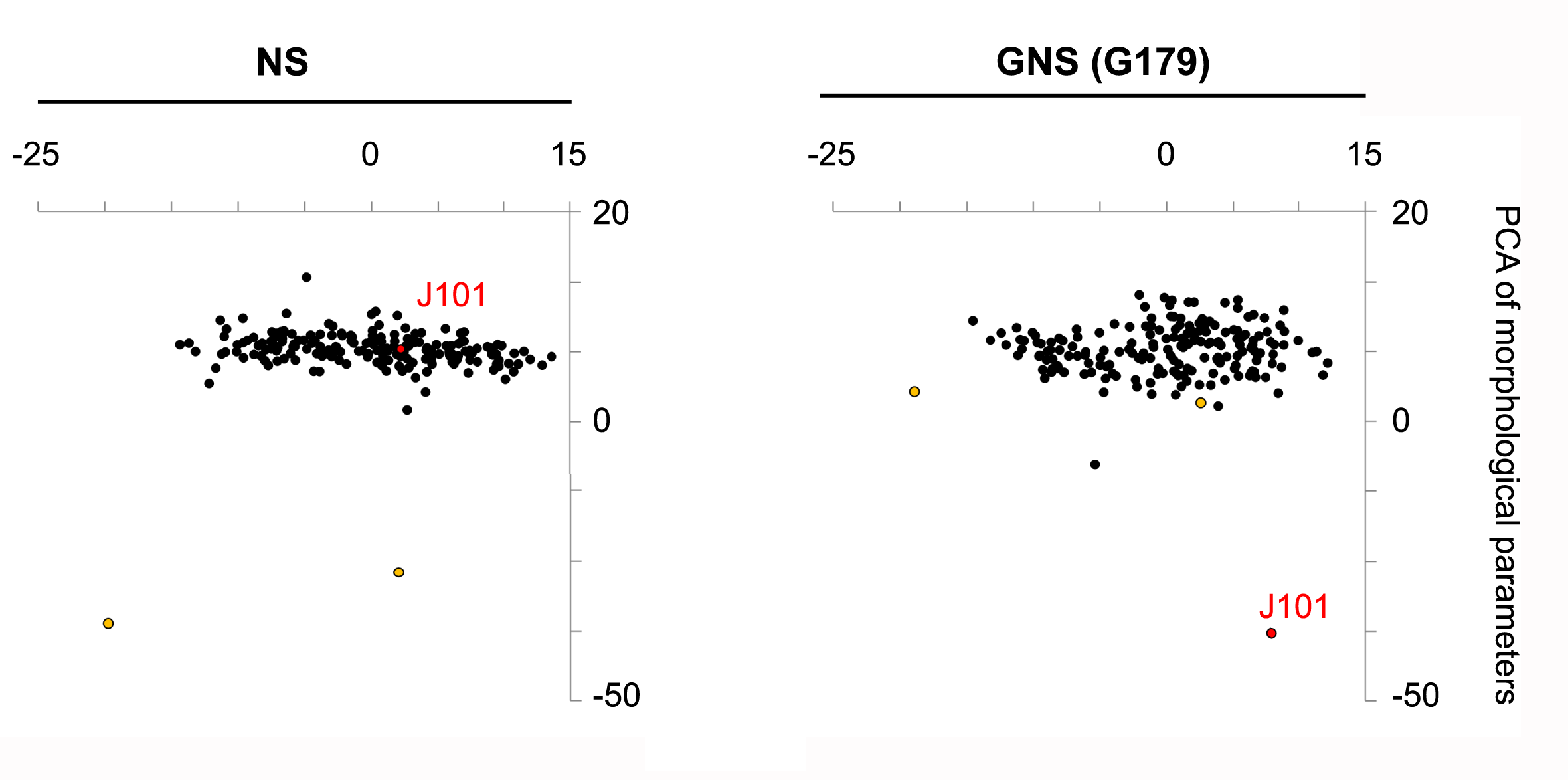

Supplement: Figure S1 — Principal component analysis (PCA) for a set of morphological parameters obtained from the kinase inhibitor screen. None of the agents in the library, including several PDGFR inhibitors, were able to induce similar mitotic phenotypes. J101 (red dots) was identified in GNS cells but not NS cells as imposing a distinct cellular morphology that was subsequently validated as mitotic arrest. The morphological features of J101-treated cells are distinct from the similar rounded morphology resulting from cell death in response to staurosporine (yellow dots). (TIF) [file pone.0077053.s001.tif]

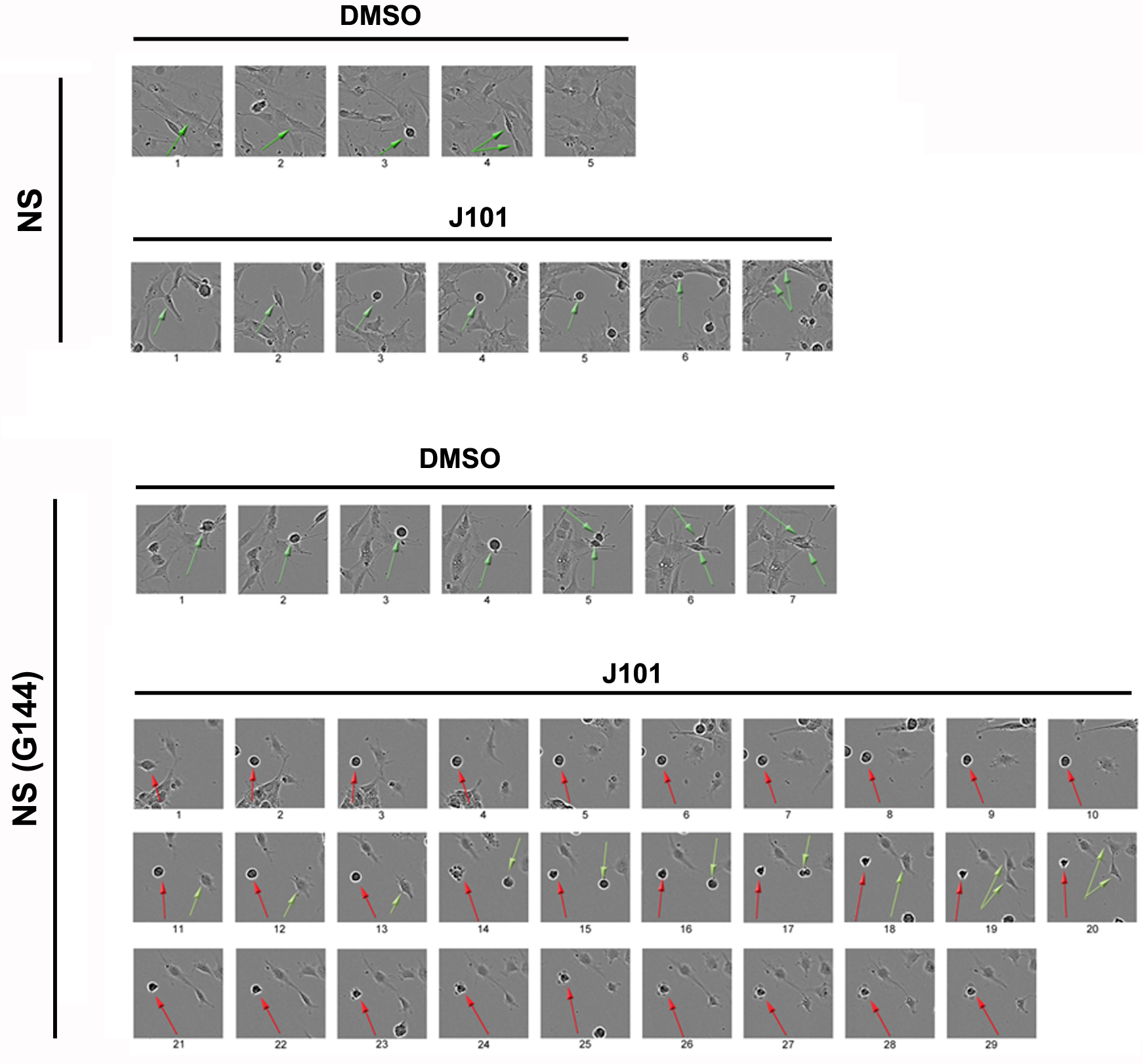

Supplement: Figure S2 — Tracking of individual cells confirms J101 treatment imposes mitotic delays that can lead to mitosis. Images were obtained at hourly intervals and manually analysed to track individual cells following treatment with DMSO or J101 (100 nM). For NS cells (top), although J101-treated cells were stalled during mitosis, this was resolved after several hours. By contrast, GNS cells (bottom panels) typically arrested at mitosis and eventually underwent apoptosis (see red arrow, frame 14). (TIF) [file pone.0077053.s002.tif]

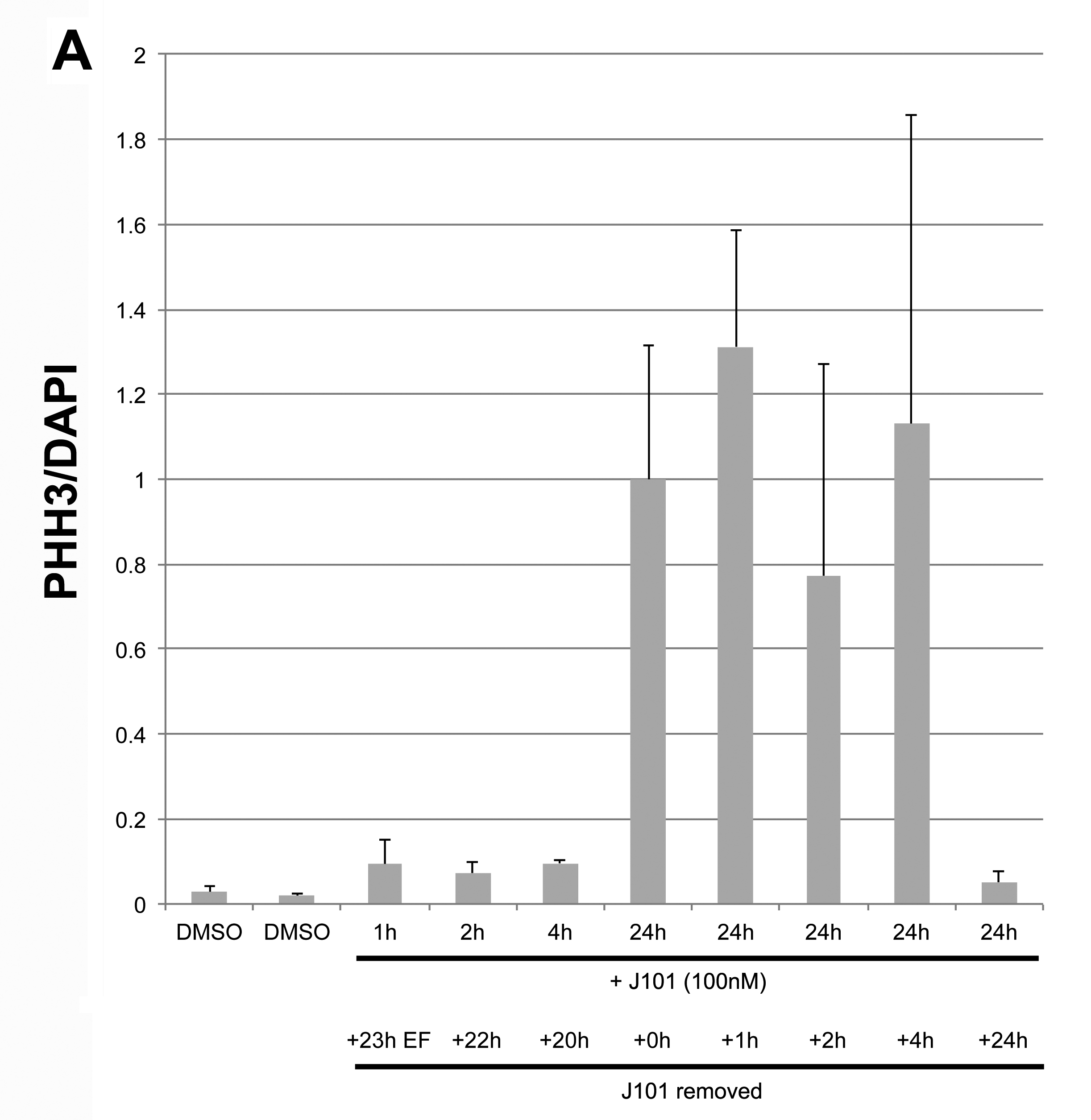

Supplement: Figure S3 — Removal of J101 from GNS cells does not enable progression through mitosis. GNS cells (G7) were treated with J101 (100 nM) for 24 h. Inhibitor was then removed 0, 2, or 4 h later and cells fixed and stained for the mitotic marker PHH3. Mitotically arrested cells did not immediately proceed through mitosis following drug removal and the majority underwent apoptosis by 24 h. (TIF) [file pone.0077053.s003.tif]

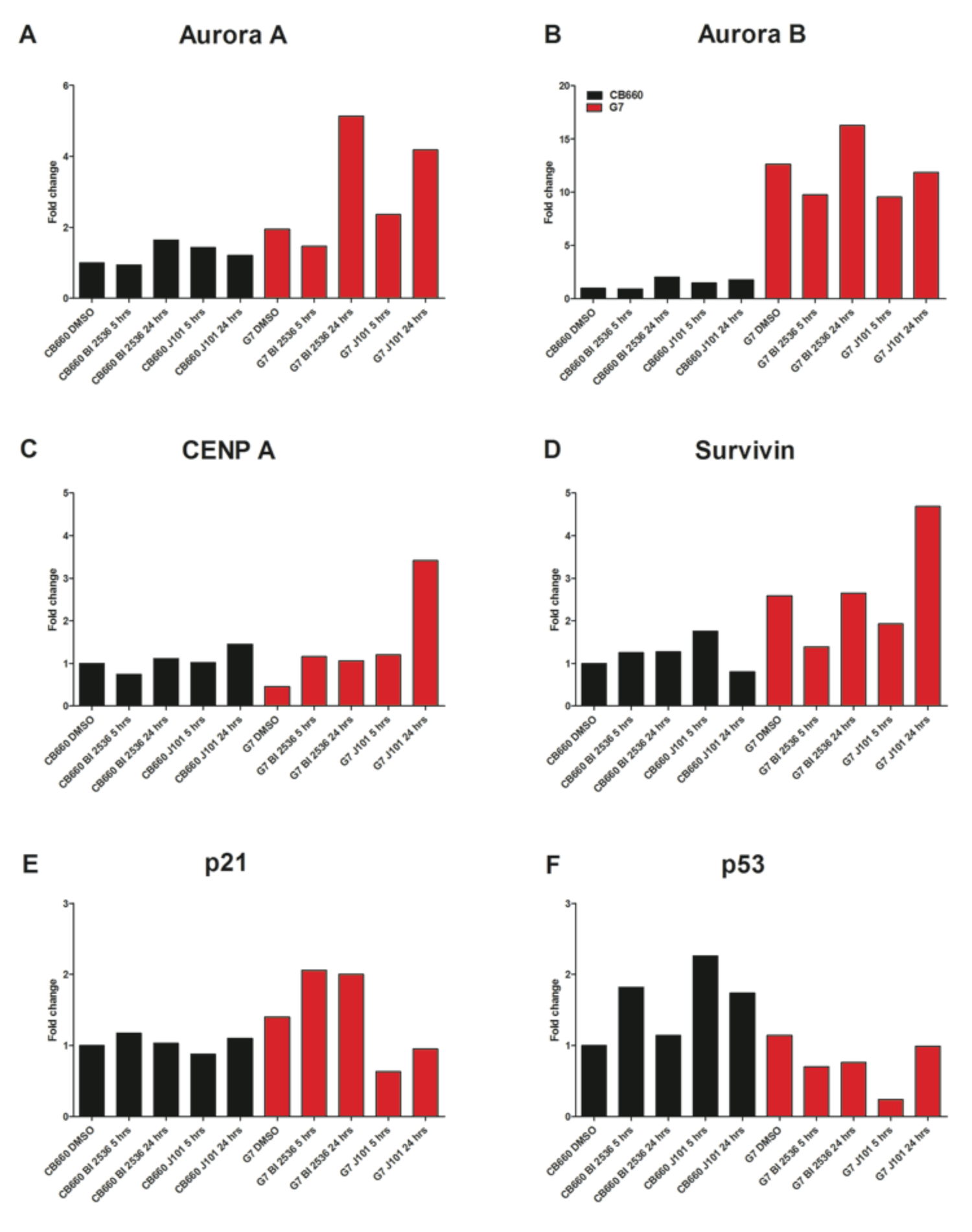

Supplement: Figure S4 — mRNA expression levels of FoxM1 downstream targets and related genes in CB660 foetal NS cells (black) and G7 GNS cells (red) as determined by qRT-PCR. mRNA was harvested after cells were treated with DMSO, BI 2536 (100 nM) and J101 (100 nM) for 5 or 24 h. Data are expressed as fold change relative to CB660 (DMSO). Values are normalised to GAPDH expression. There is no downregulation of FOXM1 transcriptional targets, suggesting the inhibitor lies downstream of FOXM1 activity. (TIF) [file pone.0077053.s004.tif]

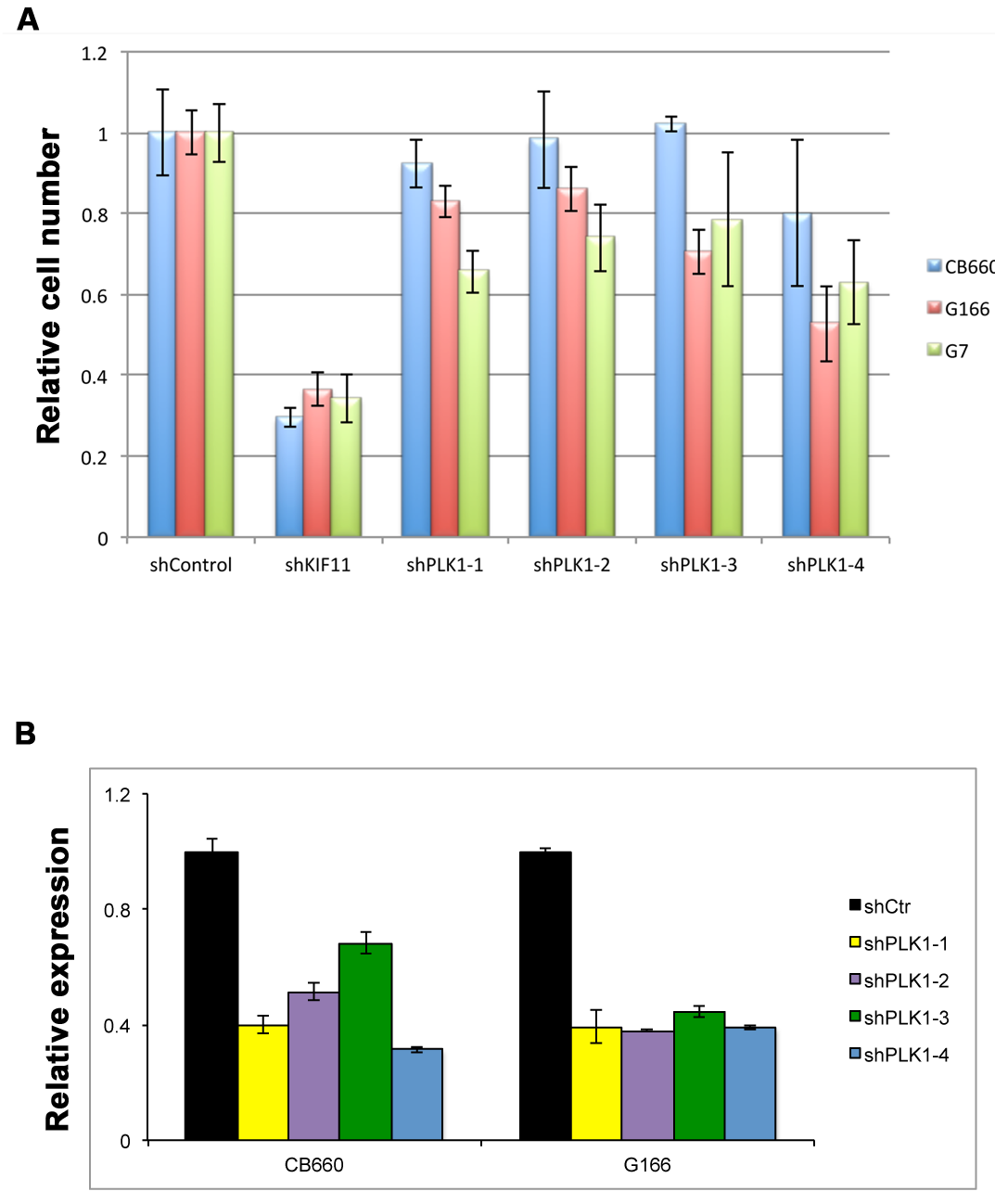

Supplement: Figure S5 — Transient knockdown of Plk1 mRNA using RNAi. (A) Four different shRNAs were tested and relative cell numbers were scored. We tested G166 and G7 as these exhibited the least and greatest response to J101 treatment, respectively. For both lines we observed a greater suppression of proliferation in GNS cells (G166 or G7) than normal NS cells. (B) qRT-PCR for Plk1 confirms Plk1 knockdown using these shRNAs. (TIF) [file pone.0077053.s005.tif]

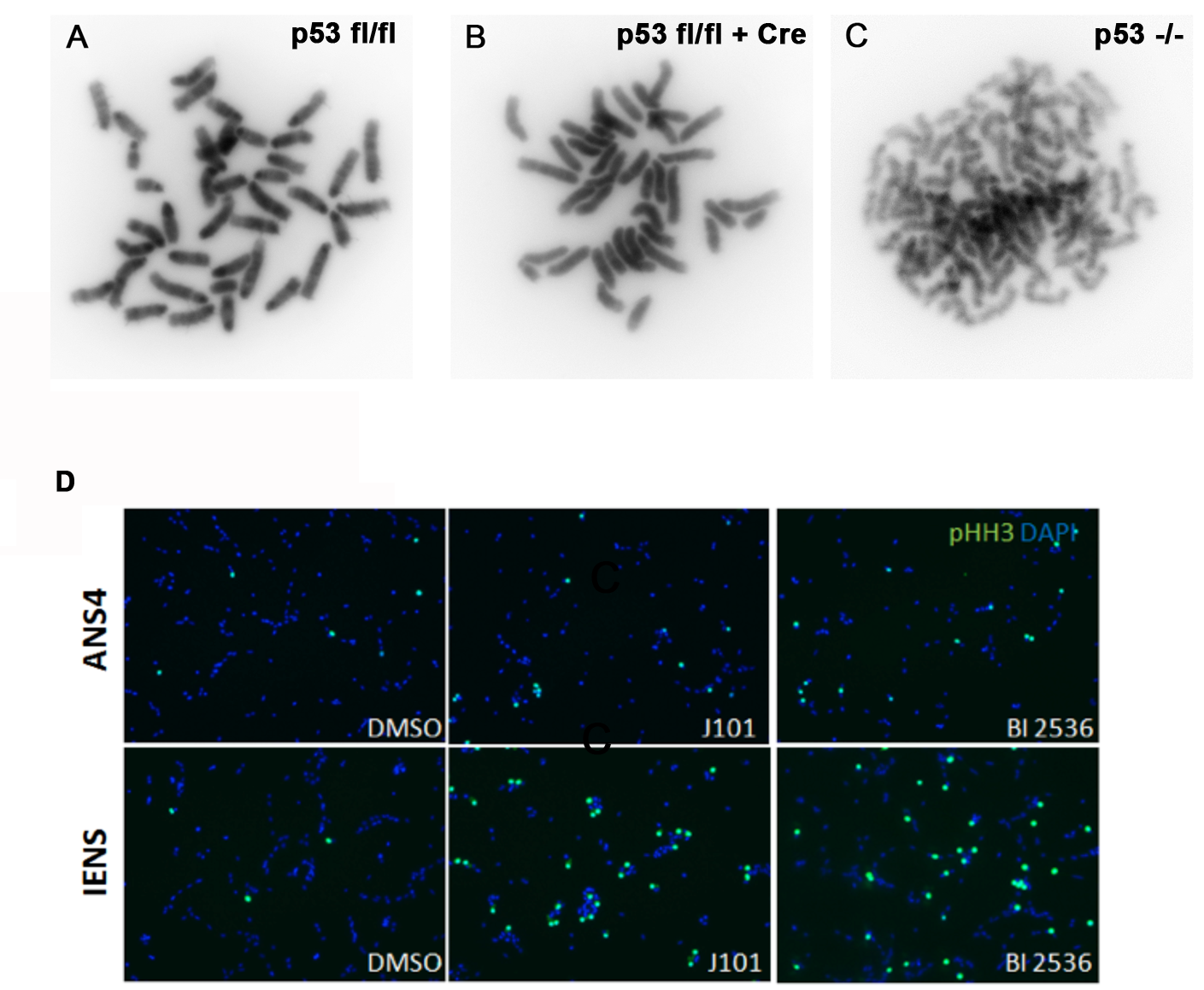

Supplement: Figure S6 — Metaphase spreads of mouse mutant NS cell lines. (A) p53fl/fl cells. (B) p53fl/fl cells transduced with CRE recombinase. (C) p53−/− cells. (D) IENS cells (INK4A/ARF−/− plus EGFRvIII over-expressing NS cells) also display greater sensitivity to Plk1 inhibitors. Genetically normal mouse NS cells (ANS4) were less sensitive than the mouse glioma NS cell (IENS) to both J101 and BI 2536 treated (100 nM each). Cells were treated for 24 h and fixed and immunostained for pHH3. DAPI nuclear counterstain (blue). Sensitivity to Plk1 inhibitors is associated with loss of p53 signalling and occurs in the absence of aneuploidy. (TIF) [file pone.0077053.s006.tif]
